# Comparison of Three Expanded-Spectrum Cephalosporin Hydrolysis Assays and the NG-Test CTX-M Multi Assay That Detects All CTX-M-Like Enzymes

**DOI:** 10.3390/diagnostics12010197

**Published:** 2022-01-14

**Authors:** Camille Gonzalez, Christian Moguet, Arnaud Chalin, Saoussen Oueslati, Laurent Dortet, Stéphanie Simon, Hervé Volland, Thierry Naas

**Affiliations:** 1 Team “Resist” UMR1184 Immunology of Viral, Auto-Immune, Hematological and Bacterial Diseases (IMVA-HB), INSERM, Faculty of Medicine, Université Paris-Saclay, CEA, LabEx LERMIT, 94270 Le Kremlin-Bicêtre, France; gonzalezcamille0405@gmail.com (C.G.); Oueslati.saoussen@gmail.com (S.O.); Laurent.dortet@aphp.fr (L.D.); 2 Bacteriology-Hygiene Unit, Assistance Publique-Hôpitaux de Paris, AP-HP Paris-Saclay, Bicêtre Hospital, 94270 Le Kremlin-Bicêtre, France; 3 Département Médicaments et Technologies pour la Santé, Université Paris-Saclay, CEA, INRAE, 91191 Gif-sur-Yvette, France; christian.moguet@cea.fr (C.M.); Stephanie.SIMON@cea.fr (S.S.); Herve.VOLLAND@cea.fr (H.V.); 4 Research and Development Department, NG Biotech, 35480 Guipry, France; a.chalin@ngbiotech.com; 5 Associated French National Reference Center for Antibiotic Resistance: Carbapenemase-Producing Enterobacteriaceae, 94270 Le Kremlin-Bicêtre, France

**Keywords:** biochemical assay, CTX-M, ESBL detection, expanded-spectrum cephalosporin hydrolysis, lateral flow immunoassay, LFIA, rapid diagnostics

## Abstract

Rapid detection of expanded-spectrum cephalosporins (ESC) hydrolysing enzymes is crucial to implement infection control measures and antibiotic stewardship. Here, we have evaluated three biochemical ESC hydrolysis assays (ESBL NDP test, β-LACTA™ test, LFIA-CTX assay) and the NG-Test^®^ CTX-M MULTI that detects CTX-M enzymes, on 93 well-characterized Gram-negative isolates, including 60 *Enterobacterales*, 21 *Pseudomonas* spp. and 12 *Acinetobacter* spp. The performances were good for all three hydrolysis assays, with the LFIA-CTX being slightly more sensitive and specific on the tested panel of isolates especially with *Enterobacterales*, without ambiguous results. This study showed that LFIA-CTX may be used for the detection of ESC hydrolysis as a competitive alternative to already available assays (β-LACTA™ test and ESBL NDP test) without any specific equipment and reduced hands-on-time. The lateral flow immunoassay NG-Test^®^ CTX-M MULTI has proven to be a useful, easy, rapid, and reliable confirmatory test in *Enterobacterales* for detection of CTX-M-type ESBLs, which account for most of the resistance mechanisms leading to ESC resistance in *Enterobacterales*, but it misses rare ESC hydrolysing β-lactamases (AmpC, minor ESBLs, and carbapenemases). Combining it with the LFIA-CTX assay would yield an assay detecting the most frequently-encountered ESBLs (CTX-M-like β-lactamases) together with ESC hydrolysis.

## 1. Introduction

The fight against infectious diseases is one of the greatest public health challenges, especially with the emergence of Multi-Drug Resistant (MDR) Gram-Negative Bacteria (GNB) [1]. The emergence of resistant bacteria has accelerated in recent years, mainly as a result of increased selective pressure. MDR gram-negative pathogens, and especially *Enterobacterales*, *Pseudomonas aeruginosa*, and *Acinetobacter baumannii*, are emerging worldwide, and are in some cases resistant to all available drugs [2,3]. β-Lactams are among the most frequently prescribed antibiotics used to treat bacterial infections, but β-lactamase-mediated resistance does not spare even the most powerful β-lactams (i.e., expanded-spectrum cephalosporins (ESCs) and carbapenems), whose activity is compromised by extended-spectrum β-lactamases (ESBLs), plasmid and hyperproducing chromosomally-encoded cephalosporinases, and carbapenemases (KPC, OXA-48, NDM). ESBLs are by far the most prevalent ESC resistance mechanism in *Enterobacterales*, with CTX-Ms representing the most prevalent ESBLs worldwide. The dissemination of these enzymes is a matter of great clinical concern given the major role of these pathogens as causes of nosocomial infections (and, for *E. coli*, also of community-acquired infections), and the major role of expanded-spectrum cephalosporins and carbapenems in the treatment of those infections [4]. The CDC has estimated that ESBL-producing *Enterobacterales* (ESBL-E) account for 19% of health care-related infections annually and that infections involving ESBL-E are also associated with increased mortality and cost of care [5]. Thus, the rapid detection of ESC hydrolysis is a critical step to guide treatments of infected patients and prevent their dissemination by implementing proper infection control measures [5].

Detection of MDR GNB relies on phenotypic approaches such as antimicrobial susceptibility, which require at least 24 h [6]. Rapid diagnostic tests have been developed to reduce the time to results from more than 24 h to only a few hours [6]. These tests are based on molecular biology approaches [6], on biochemical evidence of ESC hydrolysis, such as the home-made ESBL NDP (Nordmann–Dortet–Poirel) test [7], or the commercially available β-LACTA™ test [8] (Bio-Rad, Marne-la-Coquette, France), or on the detection of an enzyme known to hydrolyse ESCs such as the commercially available NG-Test^®®^ CTX-M MULTI (NG-Biotech, Guipry, France) [9], an easy, rapid and reliable lateral flow immunoassay (LFIAs) for CTX-M-like enzyme detection. Very recently, a research-use-only (RUO) LFIA-CTX test (NG-Biotech) combining a hydrolysis test with LFIA detection using monoclonal antibodies recognizing only the non-hydrolysed cefotaxime was developed [10]. This assay is able to detect the presence of β-lactamases that are capable of hydrolysing cefotaxime, such as hyper-producers of the chromosomally-encoded cephalosporinases (cAmpC), plasmid-encoded cephalosporinases (pAmpC), ESBLs, and some carbapenemases [10].

The aim of this study was to compare the analytical performances of the LFIA-CTX assay with those of three assays widely used for revealing ESC hydrolysis or CTX-M-type β-lactamase detection on a collection of 93 isolates possessing well-characterized β-lactam-resistance mechanisms.

## 2. Materials and Methods

### 2.1. Strain Collection

We examined 93 isolates possessing β-lactam-resistance mechanisms that have previously been well-characterized using extensive PCR approaches, or whole genome sequencing [11,12]. Thus, 60 *Enterobacterales*, 21 *Pseudomonas* spp., and 12 *Acinetobacter* spp., harbouring single or multiple β-lactamase genes (carbapenemases, ESBLs, and plasmid- or chromosome-encoded AmpCs) were used in this study. Isolates lacking significant ESC hydrolytic activity were also included as negative controls in this study. For a complete list of isolates see Supplementary Table S1.

### 2.2. The ESBL NDP Test

The ESBL NDP test, which is based on the colour change of a pH indicator upon acidification related to the hydrolysis of the ESC cefotaxime, was performed as previously described [7]. This assay detects ESC hydrolytic activity and can differentiate between ESBL producers and non-ESBL producers, as it also tests susceptibility to tazobactam (Table 1). Indeed, most ESBLs are inhibited by the addition of tazobactam, which is not the case with cephalosporinases, oxacillinases, and most carbapenemases. Roughly, three 10 µL calibrated loops (loaded to the 1/3) of bacterial colonies were resuspended in 100 µL of lysis buffer (B-PERII, Pierce, Thermo Scientific, Villebon-sur-Yvette, France) in three 1.5 mL Eppendorf tubes (labelled A, B, and C). Then, 10 μL of a concentrated tazobactam solution (40 mg/mL) were added to tube C. In tube A (internal control), 100 μL of the revealing solution containing a pH indicator (phenol red) were added. In tubes B and C (test tubes), 100 μL of an extemporaneously prepared revealing solution supplemented with cefotaxime at 6 mg/mL were added. Tubes A, B, and C were incubated at 37 °C for a maximum of 15 min [7]. The results were considered negative when all tubes remained red and interpreted as non-ESC hydrolysing and non-ESBL. When tube B and C were yellow/orange and tube A was red, the result was interpreted as ESC-hydrolysis; when tube B was yellow/orange and both tubes A and C were red, the test result was considered positive for ESBL-producing isolate; and when tube A turned yellow/red, the test result was considered noninterpretable, regardless of any colour change for tubes B and C [7].

### 2.3. LACTA™ Test

The β-LACTA™ test, which is based on a colour change upon hydrolysis of the chromogenic cephalosporin HMRZ-86 [12], was performed according to the manufacturer’s instructions [8]. The β-LACTA™ test detects ESC hydrolysing activity but cannot differentiate between ESBL production, overproduced or plasmid-encoded cephalosporinases, and/or carbapenemases [8]. A single 1 µL loop full of bacterial colonies was resuspended in a 1.5 mL microtube containing 1 drop of reagent R1 and 1 drop of reagent R2, and subsequently incubated at room temperature for 15 min, prior to visual reading of the test result. No change in colour was considered a negative result (no hydrolysis of HMRZ-86), a purple-red colour was considered a positive result, and an orange colour was considered a non-interpretable (NI) result (Table 1). For the β-LACTA™ test two separate calculations of test performances were performed, one with the NIs considered positive, and one with the NIs considered negative [8].

### 2.4. NG-Test^®^ CTX-M MULTI

The NG-Test^®^ CTX-M MULTI is a LFIA that detects all CTX-M-like enzymes, on pure isolates grown on agar plates [9]. This assay requires only one colony resuspended in 100 µL of extraction buffer and loaded on the LFIA cassette (Table 1). However, this assay is not able to detect other ESC hydrolysing β-lactamases such as hyper-producers of the chromosomally-encoded cephalosporinases (cAmpC), plasmid-encoded cephalosporinases (pAmpC), minor ESBLs and some of the carbapenemases [9].

### 2.5. LFIA-CTX Test

The recently developed RUO LFIA-CTX test, which is based on the detection of cefotaxime-hydrolysis using monoclonal antibodies that discriminate between cefotaxime and its hydrolysed products, was performed as previously described [10]. 150 μL of extraction buffer was added to a 1.5 mL Eppendorf tube containing lyophilised cefotaxime for a final concentration of 30 ng/mL, and one single colony was resuspended. After a 30 min incubation at room temperature, 100 μL were loaded on the cassette and the results were read after 10 min of migration by the naked eye [10].

### 2.6. Statistical Analysis

The sensitivity and specificity values of evaluated assays were calculated with their respective confidence intervals (95% CI) using the free software VassarStats [13].

## 3. Results

### 3.1. The ESBL NDP Test

The performances of the ESBL NDP test were similar for the three tested species, however with slightly better results in *Enterobacterales* with specificity and sensitivity of 93% and 89%, respectively (Table 2). The ESBL NDP test failed to detect in *Enterobacterales* seven ESBL producers (five CTX-Ms, one TEM-24, and one GES-6) and an overexpressed AmpC in *E. cloacae*. Similarly, three ESBL- *P. aeruginosa* isolates (SHV-2A, SHV-5, TEM-4) and two ESBL-*Acinetobacter* spp. (CTX-M-15 and VEB-1) were not detected, likely due to low levels of β-lactamase expression. In addition, an OXA-13 producing *P. aeruginosa* was missed, which could be explained by the low level of cefotaxime hydrolysis conferred by OXA-13 oxacillinase [14].

### 3.2. The β-LACTA^™^ Test

As previously shown, the detection of ESC hydrolysing activity in *Enterobacterales* using the β-LACTA^™^ test displays a good specificity (93%) and sensitivity that varies between 89% and 77% depending on whether the NIs are considered positive or negative, respectively (Table 2). Four ESBL producers were not detected (CTX-M-37, -17, -10, and GES-6 together with a plasmid-encoded cephalosporinase (CMY-136)). Even though the β-LACTA^TM^ test was not validated for *P. aeruginosa* and *A. baumannii*, we tested it on our collection of isolates [7,8]. For *P. aeruginosa* a lower specificity (80%) and sensitivity (89% and 66%) were observed. Three ESBL producers were not detected (GES-5, SHV-2a, and TEM-4). For *Acinetobacter* spp. excellent specificity (100%) and sensitivity (100–72%) were observed. As the number of tested isolates was low, especially for Acinetobacter spp., these results need to be confirmed on a larger panel of isolates. The main challenge with the β-LACTA^™^ test is the interpretation of the NI results, which represent 13 cases out of 93, which if considered as negative will result in an increase of false negative results, which significantly decreases the sensitivity in every tested species.

### 3.3. The NG-Test^®®^ CTX-M MULTI

Thus, the test performances for CTX-M detection were 100% for both specificity and sensitivity, but if ESC hydrolysis detection is considered, lower performances were observed (Table 2). Indeed, the specificity and sensitivity for the tested panel of isolates were 100% and 62%, respectively, for *Enterobacterales*, and even lower for *P. aeruginosa* and *A. baumannii*, as only a few CTX-M producers were included in the study. These values have to be put into balance with the fact that ESBLs are by far the most prevalent ESC resistance mechanism in *Enterobacterales*, with CTX-Ms representing the most prevalent ESBLs worldwide. In a recent study, CTX-M enzymes were responsible for 98% of ESC resistance in 100 consecutive ESC-resistant clinical *Enterobacterales* identified in a clinical setting in France, either from colonies or from positive blood cultures [9]. For *P. aeruginosa* and *A. baumannii*, this assay is less relevant, as CTX-M enzymes are still rare.

### 3.4. The LFIA-CTX Test

The analytic performances of the LFIA-CTX test were similar to those of the β-LACTA^TM^ test and the ESBL NDP test (Table 2). Indeed, 100% specificity was observed for all species while sensitivity was 100%, 75%, and 100% for *Enterobacterales*, *P. aeruginosa*, and *A. baumannii*, respectively (Table 2). The sensitivity for *P. aeruginosa* was comparable to those of the β-LACTA™ test and the ESBL NDP test, as the same three ESBLs producers (GES-5, SHV-2a, TEM-4) plus PME-1 producing *P. aeruginosa* were not detected. The presence of the genes in these isolates was confirmed by PCR (data not shown). Finally, the LFIA-CTX test detected cefotaxime hydrolysing enzymes efficiently in the 12 *Acinetobacter* spp. isolates tested.

## 4. Discussion

The rapid and effective detection of antibiotic resistant bacteria is a critical step for antibiotic stewardship and infection control. Despite technological improvements, the identification of pathogenic bacteria, as well as the detection of antibiotic resistance, remains complex and time-consuming, with time to results often above 24 h [6,15]. Lateral flow immunoassays (LFIAs) have proven to be useful, easy, rapid, and reliable confirmatory tests for detection of β-lactamases, especially for CTX-M-type ESBLs in gram-negatives [9,16,17]. This assay detects the presence of CTX-Ms that account for most of the resistance mechanisms leading to ESC resistance in *Enterobacterales* but misses some rare ESC hydrolysing β-lactamases (AmpC, minor ESBLs, and carbapenemases). Biochemical-based confirmatory tests evaluate the enzyme’s ability to hydrolyse ESCs. The β-LACTA™, the ESBL NDP, and the LFIA-CTX tests are well-adapted for the detection of *Enterobacterales* isolates expressing enzymes hydrolysing cefotaxime. The ESBL NDP test and β-LACTA™ test, even though displaying good analytical performances, are sometimes difficult to interpret, and need large amounts of bacteria [8]. The LFIA-CTX results are obtained with one single colony as compared to the β-LACTA™ and ESBL NDP tests, which require a 1 µL a loop and a full 10 µL calibrated loop (loaded to the 1/3), respectively. The LFIA-CTX was highly specific, with global performances close to 100%, for *Enterobacterales* but also for *Pseudomonas* spp. and *Acinetobacter* spp. isolates hydrolysing cefotaxime. Indeed, despite the natural resistance to cefotaxime by *Pseudomonas* spp. and *Acinetobacter* spp., acquired enzymatic activity could be detected. The LFIA-CTX test is slightly longer as, unlike the two other tests that require only 15 min, it needs a preincubation of 30 min incubation with cefotaxime and a 10 min migration. On the other hand, for the LFIA-CTX test and the β-LACTA™ test, incubation is performed at room temperature, while for the ESBL NDP test a 37 °C incubation is required (Table 1).

With some isolates, discrepant results were obtained among the three tests. Indeed, for SME-1-producing *S. marcescens* and a CARB-4 producing *P. aeruginosa* isolates, positive results were obtained with the β-LACTA™ test and the ESBL NDP test, but with the LFIA-CTX assay the result was negative [18,19]. SME-1, a class A carbapenemase, and CARB-4, a broad-spectrum penicillinase, are devoid of significant cefotaxime hydrolysing activity [20,21]. Thus, the results of LFIA-CTX are in agreement with the literature. These differences might be explained by the number of bacteria used for these tests. Indeed, the LFIA uses one colony, while the other tests use a 1 µL or 1/3 of a 10 µL loop full of bacteria. Similarly, the β-LACTA™ test was positive for the RTG-4 producing *A. baumannii*, whereas the LFIA-CTX and the ESBL NDP tests were negative. RTG-4 is a penicillinase (carbenicillinase or CARB) that has broadened its spectrum of hydrolysis to include cefepime and cefpirome but has no detectable cefotaxime hydrolytic activity [20].

## 5. Conclusions

This study showed that LFIA-CTX may be used for the detection of ESC hydrolysis as a competitive alternative to already available assays (β-LACTA™ test and ESBL NDP test) without any specific equipment and reduced hands-on time. The ESBL NDP test is a home-made assay that allows distinction between ESBL and other ESC hydrolysing enzyme producers. Combining the LFIA-CTX test with the already commercially available NG-Test^®®^ CTX-M MULTI would allow detection of ESC hydrolytic activity together with the most prevalent ESC-resistance mechanism in *Enterobacterales* (CTX-X-M-like β-lactamases).

## Figures and Tables

**Table 1:** Comparison of the three biochemical hydrolysis assays and the NG-Test CTX-M MULTI assay that detects all CTX-M-like enzymes.

	ESBL NDP	ß-LACTA™	LFIA-CTX	NG-Test^®®^ CTX-M MULTI
Origin Manufacturer	Home-made [7]	Commercial Bio-Rad (France) [8]	RUO device NG-Biotech (France) [10]	Commercial NG-Biotech (France) [9]
Substrate used	Cefotaxime	HMRZ-86: chromogenic cephalsporin	Cefotaxime	NA ^a^
Detection	Colour change due to acidification of the media as a consequence of hydrolysis	Colour change due to hydrolysis of the chromogenic cephalosporin (HMRZ-86)	Antibodies detecting non-hydrolysed cefotaxime	Antibodies detecting CTX-M enzymes
Hands-on time	15′	2′	2′	2′
Time to results	15′ incubation	15′ incubation	30′ hydrolysis and 10′ migration on the strip	15′ migration on the strip
Incubation Temperature	37 °C	RT ^b^	RT	RT
Bacterial matrix	3 × 10 µL loop full with 1/3 of bacteria to be tested	One 1 µL loop full of bacteria	One colony	One colony
Negative test	Red	Yellow	Absence of a band	Absence of a band
Positive test	Orange to Yellow	Red	Presence of a band	Presence of a band
Non-Interpretable		Orange		
Required reagents	Tazobactam, cefotaxime, and phenol red solutions needed to be prepared extemporaneously 3 different tubes needed/test	None (everything is provided) 1 tube needed/test	None (everything is provided in the kit) 1 tube needed/test	None (everything is provided in the kit) 1 tube needed/test
Storage	2–8 °C	2–8 °C	RT	RT
Interpretation	Ambiguous Hydrolysis of CTX Inhibition by Tazobactam (ESBL)	Ambiguous Hydrolysis of HMRZ-86	Unambiguous Hydrolysis of CTX	Unambiguous Detection of CTX-M-like enzymes

^a^ NA: not applicable; ^b^ RT: room temperature.

**Table 2:** Global performances of the β-LACTA^™^, ESBL NDP, LFIA-CTX, and NG-Test^®^ CTX-M MULTI tests on different isolates of *Enterobacterales, P. aeruginosa*, and *A. baumannii.* Values in brackets are the 95% confidence interval with alpha = 0.05.

	*Enterobacterales* (*n* = 60)		*P. aeruginosa* (*n* = 21)		*A. baumannii* (*n* = 12)		Global (*n* = 93)	
	Specificity	Sensitivity	Specificity	Sensitivity	Specificity	Sensitivity	Specificity	Sensitivity
β-LACTA™ with NI(+) ^a^	93% (66; 99)	89% (76; 95)	80% (63; 98)	89% (30; 99)	100% (5; 100)	100% (68; 100)	90% (65; 98)	90% (79; 95)
β-LACTA™ with NI(−) ^b^	93% (66; 99)	77 (63; 87)	80% (30; 99)	66% (41; 86)	100% (5; 100)	72% (39; 93)	90% (65; 98)	72% (60; 81)
ESBL NDP	93% (66; 99)	89% (76; 95)	80% (30; 99)	80% (56; 93)	100% (5; 100)	82% (48; 97)	89% (65; 98)	84% (73; 91)
LFIA-CTX	100% (72; 100)	100% (91; 100)	100% (60; 100)	69% (39; 90)	100% (6; 100)	100% (68; 100)	100% (82; 100)	94% (87; 98)
NG-Test^®®^ CTX-M MULTI (CTX-M-detection) ^c^	33/33		1/1		1/1		35/35	

^a^ Calculations with all 13 Non-Interpretable (NI) results considered positive; ^b^ calculations with all the 13 Non-Interpretable (NI) results considered negative; ^c^ numbers of detected CTX-M producers among the total CTX-M-producers.

## Data Availability

Not applicable.

## Supplementary Materials

The following supporting information can be downloaded at: https://www.mdpi.com/article/10.3390/diagnostics12010197/s1, Table S1: Detailed results for the 93 bacterial isolates studied with each tested method.
