# Supplementary material for: Comparison of Three Expanded-Spectrum Cephalosporin Hydrolysis Assays and the NG-Test CTX-M Multi Assay That Detects All CTX-M-Like Enzymes
Source: Diagnostics (Basel). 2022 Jan 14;12(1):197. doi: 10.3390/diagnostics12010197 (PMC8775164; doi:10.3390/diagnostics12010197)
Supplement: Supplementary file 1 [file diagnostics-12-00197-s001.zip › diagnostics-1544729-supplementary.pdf]

Supplemental Table S1:

COMPARISON OF THREE EXPANDED-SPECTRUM CEPHALOSPORIN HYDROLYSIS ASSAYS AND THE NG-TEST CTX-M MULTI ASSAY THAT DETECTS ALL CTX-M-LIKE ENZYMES

Camille GONZALEZ[1,2], Christian MOGUET[3], Arnaud CHALIN[4], Saoussen OUESLATI[1,2], Laurent DORTET[1,2,5], Stéphanie SIMON[3], Hervé VOLLAND[3], Thierry NAAS[1,2,5]*

Supplemental Table S1: Detailed results for the 93 bacterial isolates studied with each tested method.

#	Isolate	Species	β-lactam resistance mechanisms[a]	β-lactamase[b]	NG-Test® CTX-M MULTI	LFIA-CTX	β-LACTA™	ESBL NDP
Enterobacterales								
1	I8R8	*E. coli*	WT	None	-	-	-	-
2	D9O66	*P. mirabilis*	WT	None	-	-	-	-
3	SalR12-B2	*Salmonella* spp.	WT	None	-	-	-	-
4	SalR10-A10	*Salmonella* spp.	WT	None	-	-	-	-
5	3F7	*K. pneumoniae*	WT, Chromosome-encoded penicillinase	SHV-1	-	-	-	-
6	I10R8	*K. pneumoniae*	WT, Chromosome-encoded penicillinase	SHV-28	-	-	-	-
7	2E1	*K. pneumoniae*	WT, Chromosome-encoded penicillinase	SHV-11	-	-	-	-
8	J2R8	*C. freundii*	WT	Low level CMY	-	-	-	-
9	J3R8	*E. cloacae*	WT	Low level ACT	-	-	-	-
10	D1O40	*E. coli*	CTX-M-enzyme lacking CTX hydrolysis	CTX-M-93	positive	-	-	-
11	1I7	*S. marscecens*	Class A carbapenemase	SME-1	-	-	positive	ESBL
12	1I3	*E. cloacae*	Class A carbapenemase	IMI-1	-	-	NI[c]	-
13	1I5	*E. asburiae*	Class A carbapenemase	IMI-2	-	-	-	-
14	CNR143D4	*E. cloacae*	Class A carbapenemase	IMI-17	-	-	-	-
15	2G2	*E. cloacae*	Hyperexpressed AmpC	High level ACT	-	positive	positive	-
16	2E3	*E. coli*	Plasmid-encoded AmpC	ACC-1	-	positive	positive	ESBL and/or AMPC
17	2E4	*K. pneumoniae*	Plasmid-encoded AmpC	DHA-2	-	positive	positive	ESBL and/or AMPC
18	I4O38	*E. coli*	Plasmid-encoded AmpC	CMY-136	-	positive	-	ESBL and/or AMPC
19	EC-R4	*E. coli*	ESBL	CTX-M	positive	positive	positive	ESBL

20	3F8	*E. aerogenes*	ESBL	TEM-24	-	positive	positive	-
21	9.40	*E. coli*	ESBL	TEM-52 + CTX-M-15	positive	positive	positive	ESBL
22	E10R13	*E. cloacae*	ESBL	GES-6	-	positive	-	-
23	2E6	*E. coli*	ESBL	CTX-M-1	positive	positive	positive	ESBL
24	A6R5	*E. coli*	ESBL	CTX-M-2	positive	positive	positive	ESBL
25	11.10	*K. pneumoniae*	ESBL	CTX-M-3	positive	positive	positive	ESBL
26	11.176	*E. coli*	ESBL	CTX-M-8	positive	positive	positive	ESBL
27	11.175	*K. pneumoniae*	ESBL	CTX-M-8	positive	positive	positive	ESBL
28	11.200	*E.cloacae*	ESBL	CTX-M-9	positive	positive	positive	-
29	E8O13	*E. coli*	ESBL	CTX-M-10	positive	positive	-	-
30	11.63	*E. coli*	ESBL	CTX-M-14	positive	positive	positive	ESBL
31	16.79	*E. coli*	ESBL	CTX-M-15	positive	positive	positive	ESBL
32	16.29	*K. pneumoniae*	ESBL	CTX-M-15	positive	positive	positive	ESBL
33	9.17	*E.cloacae*	ESBL	CTX-M-15	positive	positive	positive	ESBL
34	11.204 CAP	*C. freundii*	ESBL	CTX-M-15	positive	positive	positive	ESBL
35	16.60	*E. coli*	ESBL	CTX-M-17	positive	positive	-	-
36	G1R4	*K. pneumoniae*	ESBL	CTX-M-18	positive	positive	positive	ESBL
37	G2R4	*K. pneumoniae*	ESBL	CTX-M-19	positive	positive	positive	ESBL
38	Red-93	*E. coli*	ESBL	CTX-M-24	positive	positive	NI	ESBL
39	10.73	*E. coli*	ESBL	CTX-M-27	positive	positive	NI	ESBL
40	10.64	*E. coli*	ESBL	CTX-M-32	positive	positive	positive	ESBL
41	E3R12	*E. coli*	ESBL	CTX-M-37	positive	positive	-	ESBL
42	Red 88	*E. coli*	ESBL	CTX-M-55	positive	positive	positive	ESBL
43	10.231	*E. coli*	ESBL	CTX-M-57	positive	positive	positive	ESBL
44	Red83A	*E. coli*	ESBL	CTX-M-65	positive	positive	positive	ESBL
45	11.110	*P. mirabilis*	ESBL	CTX-M-71	positive	positive	positive	ESBL

46	16.48	*E. coli*	ESBL	CTX-M-82	positive	positive	NI	ESBL
47	16.69	*E. coli*	ESBL	CTX-M-101	positive	positive	positive	ESBL
48	1F1	*E. coli*	Class A carbapenemase	KPC-2	-	positive	positive	ESBL and/or AMPC
49	3F2	*K. pneumoniae*	Class A carbapenemase +ESBL	KPC-3 + CTX-M	positive	positive	positive	ESBL and/or AMPC
50	1A1	*E. coli*	Class B carbapenemase	NDM-1	-	positive	NI	ESBL and/or AMPC
51	CNR190E6	*E. coli*	Class B carbapenemase + ESBL	NDM-19 + CTX-M	positive	positive	positive	ESBL and/or AMPC
52	1C3	*E. coli*	Class B carbapenemase + Plasmid-encoded AmpC	VIM-1 + CMY-13	-	positive	-	ESBL and/or AMPC
53	1C9	*K. pneumoniae*	Class B carbapenemase + ESBL	VIM-1 + SHV-5	-	positive	NI	ESBL and/or AMPC
54	1D9	*E. coli*	Class B carbapenemase	IMP-1	-	positive	positive	ESBL and/or AMPC
55	1E5	*K. pneumoniae*	Class B carbapenemase	IMP-8	-	positive	positive	ESBL and/or AMPC
56	2J3	*K. pneumoniae*	Class D carbapenemase + ESBL	OXA-163 + CTX-M	positive	positive	positive	ESBL and/or AMPC
57	CNR172D10	*E. coli*	Class D carbapenemase	OXA-484	-	positive	positive	ESBL and/or AMPC
58	2J5	*S. marcescens*	OXA-48-like enzyme hydrolysing ESCs	OXA-405	-	positive	positive	ESBL and/or AMPC
59	2A1	*E. coli*	Class D carbapenemase + ESBL	OXA-48 + CTX-M-15	positive	positive	positive	ESBL and/or AMPC
60	3G8	*K. pneumoniae*	Class D carbapenemase + ESBL	OXA-370 + CTX-M-9	positive	positive	positive	ESBL and/or AMPC
Pseudomonas spp								
61	G7R1	*P. putida*	WT, Basal AmpC	Low level AMPC	-	-	-	-
62	G5R8	*P. aeruginosa*	WT, Basal AmpC	Low level AMPC	-	-	-	-
63	carba1A8	*P. aeruginosa*	Efflux (Mex C/D-OprJ)	Low level AMPC	-	-	-	-
64	carba1A9	*P. aeruginosa*	Efflux (Mex A/B-OprM)	Low level AMPC	-	-	-	-

65	E8R5	*P. aeruginosa*	Plasmid-encoded penicillinase	CARB-4	-	-	positive	ESBL
66	carba1H2	*P. aeruginosa*	Acquired oxacillinase	OXA-32	-	-	NI	-
67	D4O5	*P. aeruginosa*	Acquired oxacillinase	OXA-13	-	-	positive	-
68	carba1F1	*P. aeruginosa*	Class B carbapenemase	AIM-1	-	-	positive	ESBL and/or AMPC
69	carba1F7	*P. aeruginosa*	ESBL	PME-1	-	-	positive	ESBL
70	pyo ctrl E6	*P. aeruginosa*	ESBL	PER-1	-	positive	positive	ESBL and/or AMPC
71	carba1F8	*P. aeruginosa*	ESBL	CTX-M-2	positive	positive	positive	ESBL and/or AMPC
72	F1O26	*P. aeruginosa*	ESBL	SHV-2a	-	positive	NI	-
73	F1R7	*P. aeruginosa*	ESBL	SHV-5	-	-	positive	-
74	F2R7 ou C8O5	*P. aeruginosa*	ESBL	TEM-4	-	-	-	-
75	F10O35	*P. stutzeri*	Class B carbapenemase	DIM-1	-	positive	positive	ESBL and/or AMPC
76	carba1B2	*P. aeruginosa*	Class A carbapenemase	GES-5	-	-	-	ESBL
77	Carba1A4	*P. aeruginosa*	Class A carbapenemase	KPC-2	-	positive	positive	ESBL and/or AMPC
78	carba1E2	*P. aeruginosa*	Class B carbapenemase	NDM-1	-	positive	NI	-
79	C3O51	*P. aeruginosa*	Class B carbapenemase	VIM-2	-	positive	NI	ESBL and/or AMPC
80	carbaD2	*P. stutzeri*	Class B carbapenemase	IMP-1	-	positive	positive	ESBL and/or AMPC
81	carba2B7	*P. aeruginosa*	Class B carbapenemase	IMP-26	-	positive	positive	ESBL and/or AMPC
A. baumannii								
82	ctrl A9	*A. baumannii*	Acquired penicillinase	RTG-4	-	-	positive	-
83	ctrlB3	*A. baumannii*	Overexpressed AmpC	High level ADC	-	positive	positive	-
84	carba1A10	*A. baumannii*	Overexpressed AmpC+ OXA	High level ADC + OXA-21 (penicillinase)	-	positive	positive	ESBL and/or AMPC
85	ctrlA7	*A. baumannii*	ESBL	PER-1	-	positive	positive	ESBL and/or AMPC
86	ctrlH9	*A. baumannii*	ESBL	VEB-1	-	positive	NI	-
87	H10R8	*A. baumannii*	ESBL	CTX-M-15	positive	positive	NI	-

88	F5O22	*A. baumannii*	Class B carbapenemase	SIM-1	-	positive	NI	-
89	metalloI2	*A. baumannii*	Class B carbapenemase	IMP-4	-	positive	positive	ESBL and/or AMPC
90	OXA-58 J3	*A. baumannii*	Class D carbapenemase	OXA-253	-	positive	positive	ESBL and/or AMPC
91	OXA-58 A8	*A. baumannii*	Class D carbapenemase	High level ADC + OXA-58	-	positive	-	-
92	metalloB2	*A. baumannii*	Class A carbapenemase	GES-11	-	positive	positive	ESBL and/or AMPC
93	mG7	*A. baumannii*	Class A carbapenemase	GES-14	-	positive	positive	ESBL

[a] β-lactam resistance mechanisms: corresponds to the phenotype conferred by the main ß-lactamase encountered in the isolate.

b β-lactamase content. When no acquired enzyme, the level of expression of naturally encoded enzymes is indicated. Hen acquired enzymes are present, only the latter zre indicated.

[c]NI: Non-Interpretable
